# Probiotic Therapy as an Adjuvant in the Treatment of Periodontal Disease: An Innovative Approach

**DOI:** 10.3390/medicina61010126

**Published:** 2025-01-14

**Authors:** Liliana Sachelarie, Ioana Scrobota, Ioana Romanul, Raluca Iurcov, Georgiana Ioana Potra Cicalau, Liana Todor

**Affiliations:** 1 Department of Preclinical Discipline, Faculty of Medicine, Apollonia University, 700511 Iasi, Romania; 2 Department of Dental Medicine, Faculty of Medicine and Pharmacy, University of Oradea, 10 1st Decembrie Street, 410073 Oradea, Romania; ioana_romanul@uoradea.ro (I.R.); riurcov@uoradea.ro (R.I.); cicalau.georgiana@uoradea.ro (G.I.P.C.); liana.todor@uoradea.ro (L.T.)

**Keywords:** probiotics, periodontal therapy, oral microbiota

## Abstract

*Background and Objectives*: Periodontal inflammation, often linked to oral microbiota dysbiosis dominated by pathogenic bacteria, remains a significant challenge in periodontitis management. Traditional periodontal therapies primarily reduce the bacterial load but fail to restore the microbiota balance. Probiotics offer a promising therapeutic adjunct with their ability to enhance beneficial bacteria. This study investigates the effects of probiotics on the oral microbiota, inflammatory markers (IL-1β, TNF-α), and clinical parameters (gingival index, bleeding index, and periodontal pocket depth). *Materials and Methods*: In this pilot study, 80 patients with moderate-to-severe periodontitis were assigned to two groups. Group A received standard periodontal therapy (non-surgical periodontal therapy (NSPT)) with probiotic supplementation (*Lactobacillus reuteri*, 2 × 10⁹ CFU daily for 8 weeks), and Group B received standard treatment with a placebo. Microbiological changes were assessed via quantitative PCR, while inflammatory markers (IL-1β, TNF-α) were analyzed using ELISA. Clinical parameters, including the gingival index (GI), bleeding index (BI), and periodontal pocket depth (PPD), were measured at baseline (T0), 4 weeks (T1), and 8 weeks (T2) using standardized methods. *Results*: Probiotic therapy (Group A) significantly reduced the pathogenic bacteria and increased the beneficial bacteria levels compared to the placebo (*p* < 0.01). Inflammatory markers decreased by 37% (IL-1β) and 42% (TNF-α), while clinical parameters improved, with reductions in the gingival and bleeding indices (−1.5, −1.3) and a 2 mm decrease in the periodontal pocket depth (*p* < 0.01). *Conclusions*: Probiotics, as an adjunct to periodontal therapy, effectively restore the microbiota balance, reduce inflammation, and improve clinical outcomes in periodontitis.

## 1. Introduction

Periodontal disease is a widespread chronic inflammatory condition affecting the supporting tissues of the teeth. While the global prevalence of periodontal disease, including mild, moderate, and severe forms, is estimated to be over 50% of the adult population, severe periodontitis affects approximately 11% of the world’s population and is a leading cause of tooth loss globally [1].

Microbiological dysbiosis, characterized by the overgrowth of pathogenic bacteria such as *Porphyromonas gingivalis* and *Aggregatibacter actinomycetemcomitans,* triggers an exaggerated host inflammatory response through virulence factors, leading to tissue destruction [2].

The red complex bacteria, including *Porphyromonas gingivalis*, *Tannerella forsythia*, and *Treponema denticola*, are considered key pathogens in the development of periodontitis due to their potent virulence factors and ability to evade host defenses [1,2,3]. Alongside *Aggregatibacter actinomycetemcomitans*, these microorganisms are major contributors to periodontal inflammation and tissue destruction [2]. Beneficial bacteria, such as *Lactobacillus* spp. and *Bifidobacterium* spp., are essential in maintaining microbial homeostasis and counteracting pathogenic colonization [3].

Furthermore, the exaggerated activation of the immune system in the presence of these bacteria leads to the release of pro-inflammatory cytokines, such as IL-1β and TNF-α, aggravating the inflammatory process. Periodontitis is characterized by an excessive host inflammatory response to oral microbiota dysbiosis. Key pro-inflammatory cytokines involved in this process include interleukin-1 beta (IL-1β) and tumor necrosis factor-alpha (TNF-α), which are elevated in the periodontal tissues and contribute to tissue destruction and bone resorption [2,3]. This vicious cycle between infection and inflammation not only accelerates local damage but can also have systemic implications, being associated with diseases such as diabetes, cardiovascular disease, and other chronic inflammatory conditions [3].

Understanding how oral microbiota dysbiosis contributes to the initiation and progression of periodontal disease is essential in developing effective therapeutic strategies to reduce the bacterial load and restore the microbiological balance. Conventional mechanical therapies, such as non-surgical periodontal therapy (NSPT), effectively reduce the bacterial load. Non-surgical periodontal therapy (NSPT) includes the thorough removal of the supra- and subgingival biofilm and calculus using ultrasonic and hand instruments. However, they often fail to restore the natural balance of the oral microbiota, which is essential for long-term periodontal health [3]. These procedures are considered standard in treating periodontal disease, removing bacterial deposits, and reducing inflammation. Still, they do not directly influence the diversity or proportion of beneficial microorganisms, which is essential for long-term oral health [2].

There has been an increasing emphasis on the use of complementary approaches to supplement traditional mechanical treatments. Among these approaches, probiotics have attracted the attention of researchers and clinicians due to their potential to support the restoration of the microbiological balance [3,4]. Probiotics are live microorganisms that, when administered in adequate amounts, confer benefits to the host, helping to reduce the colonization of pathogenic bacteria and promote the growth of beneficial bacteria in the oral cavity [4].

Probiotics can significantly support conventional treatments through competition for nutrients and space, the direct inhibition of pathogens, and the regulation of the host immune response [5]. They reduce the severity of inflammation and can also contribute to preventing periodontal disease relapses, providing an integrative approach to its management. The introduction of probiotics into periodontal treatment thus represents an innovative direction, potentially significantly improving long-term clinical outcomes [5].

According to the World Health Organization, probiotics are live microorganisms that confer health benefits to the host when administered in adequate amounts. Probiotics can enhance oral health in periodontal disease by modulating the microbiota balance and reducing inflammation [4]. In dentistry, probiotics may act by increasing beneficial bacteria such as *Lactobacillus* spp. and *Bifidobacterium* spp. and possibly by regulating the inflammatory response to reduce the levels of pro-inflammatory cytokines such as IL-1β and TNF-α [5,6].

Preliminary studies suggest that *Lactobacillus reuteri*, a commonly used probiotic strain, may benefit oral health by reducing inflammation and the periodontal pocket depth [7]. This probiotic strain effectively modulates the oral microbiota, contributing to reductions in the levels of pathogenic bacteria, such as *Porphyromonas gingivalis*, and the stimulation of beneficial bacteria. Through mechanisms such as the inhibition of pathogenic bacterial biofilms and reductions in pro-inflammatory cytokine levels, *Lactobacillus reuteri* has the potential to support periodontal tissue healing and improve clinical outcomes [4,7,8,9].

However, the clinical evidence available is limited, with most studies being small and with short follow-up periods. This makes it difficult to fully assess the long-term benefits of administering this probiotic strain. Although the existing data are promising, they highlight the need for more extensive studies to validate the observed effects and to establish doses, the optimal duration of treatment, and possible effective combinations of probiotics.

Validation through robust studies is essential in integrating probiotics into standard therapeutic guidelines for the treatment of periodontal disease, thus contributing to developing more comprehensive and personalized strategies in patient care.

This study aims to evaluate the impact of probiotic therapy on the oral microbiota and periodontal inflammation. By analyzing changes in the levels of beneficial and pathogenic bacteria, inflammatory mediators, and clinical parameters, this study aims to contribute to understanding the role of probiotics as an adjuvant in treating periodontitis.

## 2. Materials and Methods

### 2.1. Study Design

This pilot study was designed to evaluate the potential effects of probiotic therapy on the oral microbiota and periodontal inflammation in patients with moderate-to-severe periodontitis. It was conducted at the Apollonia University Dental Center and approved by the Ethics Committee on 30 November 2023 at Apollonia University, Iasi, Romania. It was conducted in accordance with the CONSORT guidelines to ensure clarity and replicability in clinical trial reporting [10].

**Figure 1:**
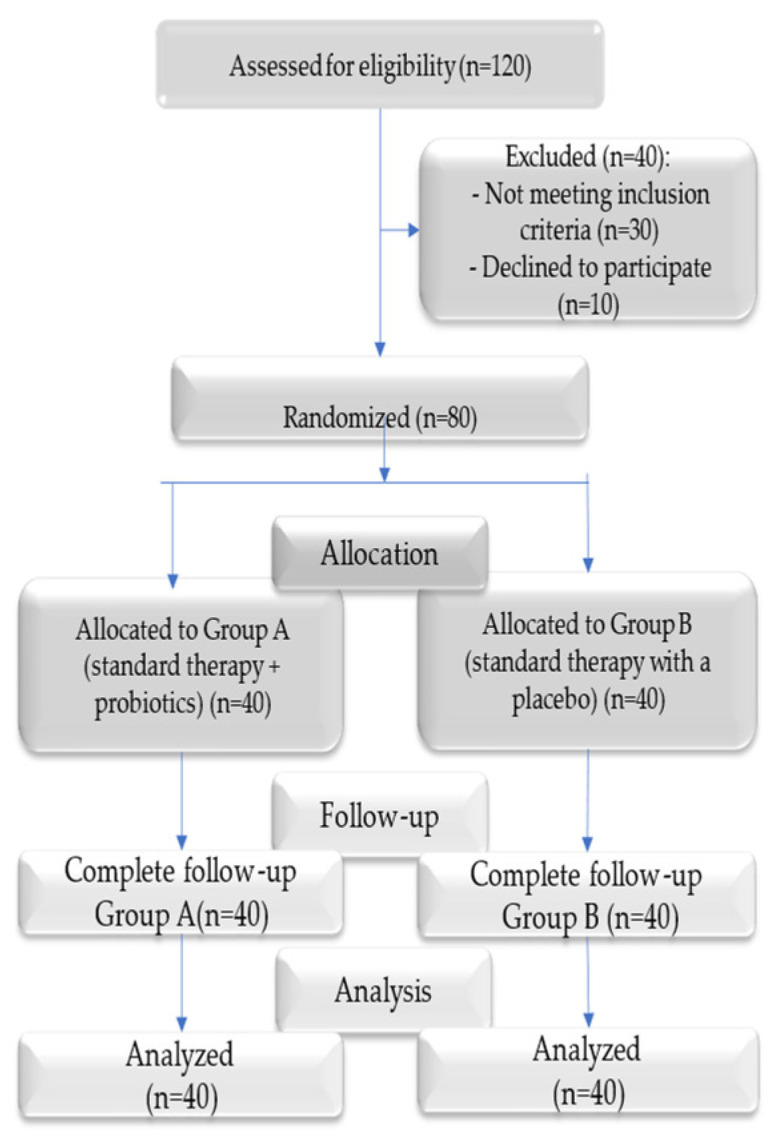
CONSORT flow diagram.

The CONSORT flow diagram in Figure 1 illustrates the selection and analysis process applied to the study participants.

This randomized, double-blind, placebo-controlled study aimed to evaluate the efficacy of probiotic supplementation as an adjunct to standard periodontal therapy in patients with moderate-to-severe periodontitis. A total of 80 participants were randomly assigned to two groups: Group A (probiotic group) and Group B (placebo group). Randomization was performed using a computer-generated sequence, and an independent pharmacist ensured allocation concealment. Both groups underwent standard periodontal therapy, including supragingival scaling and subgingival root planing. Group A received a daily capsule containing *Lactobacillus reuteri* (2 × 10⁹ CFU), while Group B received an identical placebo capsule for 8 weeks.

The parameters assessed included the gingival index (GI), which reflects the severity of gingival inflammation based on color, consistency, and bleeding on probing, and the bleeding index (BI), which quantifies bleeding occurrence as a response to probing.

The gingival index (GI) used in this study is based on the method proposed by Löe and Silness (1963/1964) [11]. The bleeding index (BI) used in this study was determined using the method developed by Ainamo and Bay (1975), which measures the occurrence of bleeding upon probing and determines the percentage of sites with bleeding [12]. Both indices were measured using standardized periodontal probes and scoring systems, ensuring accurate and reproducible assessments of gingival health. Additionally, the periodontal pocket depth (PPD) was recorded to assess tissue destruction, and the healing response over the study period was assessed at baseline (T0) and 8 weeks post-treatment (T2). Periodontal parameters were assessed by probing six sites per tooth: mesiobuccal, buccal, distobuccal, mesiolingual, lingual, and distolingual.

In this study, only T0 (baseline) and T2 (final timepoint, 8 weeks) were included in the final analysis to emphasize the differences between the intervention’s start and end points. T1 (4 weeks) was not included as it represented an intermediate phase.

Microbiological samples were collected at the same time points and analyzed using quantitative PCR to evaluate the levels of key bacterial species. Subgingival plaque samples were collected using sterile curettes from the deepest periodontal pockets (>5 mm) at baseline (T0) and after 8 weeks (T2). The samples were transferred to sterile vials containing reduced transport fluid and stored at 4 °C until transport to the laboratory within 24 h. Quantitative PCR was conducted to quantify bacterial species (*Porphyromonas gingivalis, Aggregatibacter actinomycetemcomitans, Lactobacillus* spp., *Bifidobacterium* spp.). The study followed ethical guidelines, and all participants provided informed consent before enrollment. Commercially available ELISA kits were used to quantify the IL-1β and TNF-α levels. The assay was performed according to the manufacturer’s protocol.

The inclusion criteria included a diagnosis of periodontitis based on the 2017 Workshop on Periodontology, characterized by clinical attachment loss (CAL) ≥ 3 mm at ≥2 non-adjacent teeth, a probing depth (PD) ≥ 5 mm in at least two areas, and bleeding on probing (BOP). Patients also presented with a gingival index (GI) ≥ 2, indicating moderate-to-severe gingival inflammation. The inclusion criteria did not include a specific cut-off for the remaining teeth.

The exclusion criteria included patients with systemic diseases affecting the periodontium (e.g., uncontrolled diabetes, autoimmune diseases), antibiotic or probiotic treatment occurring less than 3 months before the study, pregnancy or breastfeeding, and periodontal therapy in the last 6 months.

Both the patients and investigators were blinded to the type of intervention.

In this study, the participants were blinded to their group assignments, as the probiotic and placebo capsules were identical in appearance, size, and taste. The investigators conducting the clinical assessments, including the gingival index, bleeding index, and periodontal pocket depth, were also blinded to the group allocations to ensure unbiased evaluations. Additionally, the statistician responsible for data analysis was blinded, working exclusively with coded datasets, without access to information about the intervention groups. This rigorous double-blind approach ensured objectivity throughout the study, enhancing the reliability of the findings.

### 2.2. Assessments Performed

Assessments were performed at baseline (T0) before treatment and 8 weeks (T2) after treatment. For this study, only T0 and T2 were included in the final analysis to focus on the baseline and endpoint differences.

All participants completed the study, without any discontinuations or losses during the follow-up period, ensuring the inclusion of the entire randomized sample (*n* = 80) in the final statistical analysis.

### 2.3. Statistical Analysis

The statistical analysis was performed using paired t-tests for intra-group comparisons and repeated-measures ANOVA for between-group comparisons, with a significance level set at *p* < 0.01.

## 3. Results

### 3.1. Baseline Characteristics

Table 1 presents the baseline characteristics of the Group A (Probiotics) and Group B (Placebo) participants. In this study, the participants in Group A ranged in age from 39 to 51 years (mean age: 45 ± 6), while the participants in Group B ranged from 41 to 51 years (mean age: 46 ± 5). The data include demographic information such as the percentage of females and males, the mean age, and clinical parameters including the gingival index, the periodontal pocket depth, bleeding on probing, and the presence of pathogenic bacteria. These metrics were similar between the two groups, ensuring comparability at the study’s start.

### 3.2. Group A (Standard Therapy + Probiotics)

The gingival index (GI) showed a significant reduction from baseline (T0) to the study endpoint (T2), with a mean decrease of 1.83 points (t = 16.32, *p* < 0.0001), as illustrated in Figure 1 and detailed in Table 2. Similarly, the periodontal pocket depth (PPD) demonstrated a marked reduction of 2.42 mm between T0 and T2 (t = 43.55, *p* < 0.0001).

**Figure 2:**
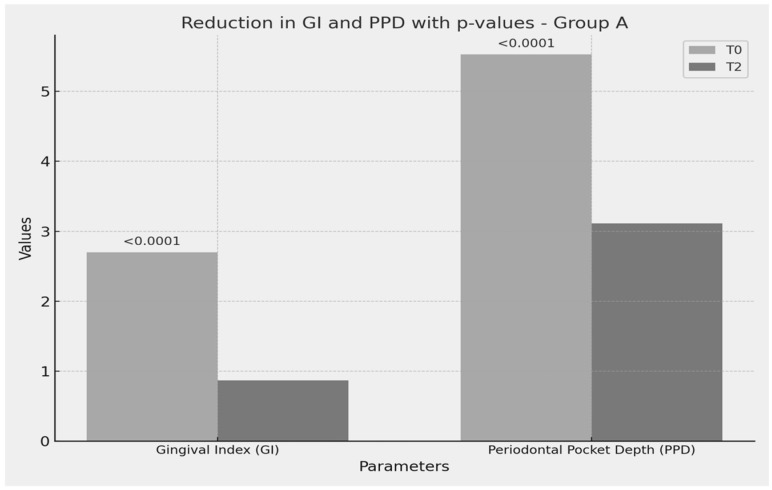
Reduction in GI and PPD with *p*-values-group A.

Figure 2 illustrates the significant reductions in the gingival index (GI) and periodontal pocket depth (PPD) observed within Group A, which received standard periodontal therapy combined with probiotics. These results highlight the enhanced therapeutic effect of *Lactobacillus reuteri* supplementation in improving clinical periodontal parameters.

The GI in Group A decreased significantly from baseline (T0) to the end of the study (T2), with a mean reduction of 1.53 points (*p* < 0.0001). The PPD reduction of 2.42 mm observed in Group A from T0 to T2 (*p* < 0.0001) demonstrates enhanced tissue healing. Figure 2 demonstrates the significant improvements in GI and PPD achieved with probiotic supplementation in Group A.

### 3.3. Group B (Standard Therapy with a Placebo)

The gingival index (GI) showed a significant reduction between T0 and T2, with a mean decrease of 0.84 points and a statistical value of t = 18.12, *p* < 0.0001 (Table 3).

The periodontal pocket depth (PPD) was also significantly reduced between T0 and T2, with a mean decrease of 0.41 mm and a statistical value of t = 11.09, *p* < 0.0001.

**Figure 3:**
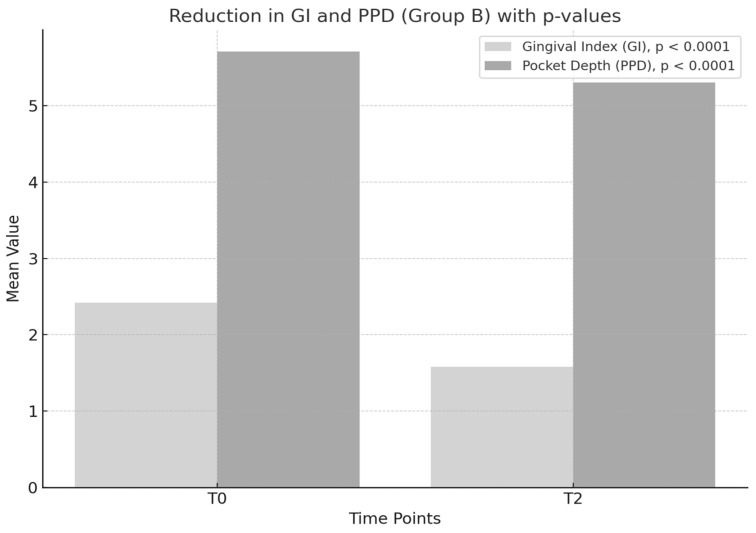
Reduction in GI and PPD with *p*-values—Group B.

The data presented in Figure 3 illustrate the changes in the gingival index (GI) and periodontal pocket depth (PPD) within Group B, which received standard periodontal therapy combined with a placebo. While the reductions in both parameters were statistically significant (*p* < 0.01), the magnitude of the change was notably smaller compared to Group A, which received probiotics in addition to standard therapy.

The mean gingival index (GI) in Group B decreased from baseline (T0) to the study endpoint (T2), indicating improved gingival inflammation. The reduction of 0.84 points, although statistically significant (*p* < 0.01), suggests a moderate therapeutic effect of the standard treatment.

The absence of probiotic supplementation likely limited the ability to achieve more substantial improvements, as the microbiota balance was not actively modulated.

The mean PPD reduction of 0.41 mm from T0 to T2 reflects the mechanical effectiveness of scaling and root planing in removing bacterial deposits and reducing inflammation. However, this reduction was considerably smaller than that observed in Group A, highlighting standard therapy alone’s limited tissue healing effect on periodontal tissues.

### 3.4. Between-Group Analysis (Repeated-Measures ANOVA)

The gingival index (GI) showed significant differences between Groups A and B at the final time point (T2), with F = 58.54, *p* < 0.0001. Group A demonstrated a more pronounced reduction in gingival inflammation compared to Group B (Table 4).

The periodontal pocket depth (PPD) also exhibited significant differences between the groups at T2, with F = 2820.31, *p* < 0.0001. The reduction in the periodontal pocket depth was notably greater in Group A than in Group B (Table 4).

**Figure 4:**
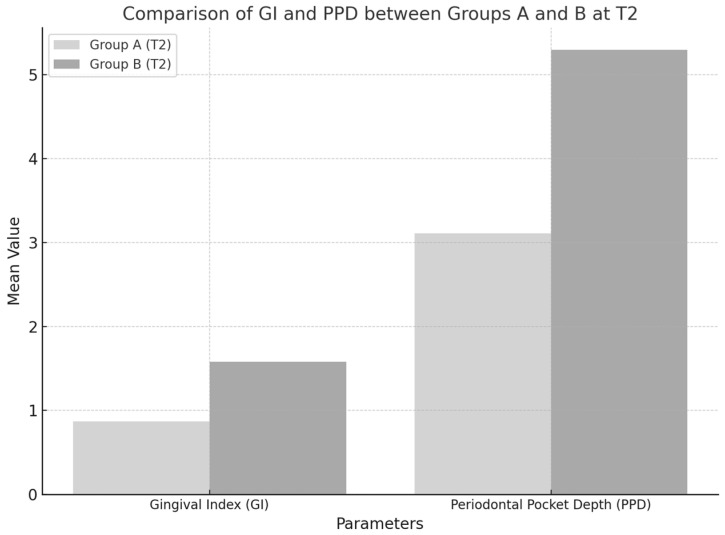
Comparison of GI and PPD between Groups A and B at T2.

Figure 4 illustrates the differences in the gingival index (GI) and periodontal pocket depth (PPD) between Group A (standard therapy + probiotics) and Group B (standard therapy + placebo) at the end of the study (T2). The data demonstrate clear advantages in terms of clinical outcomes for participants who received probiotic supplementation, supporting the hypothesis that probiotics enhance the effectiveness of standard periodontal treatment.

At T2, Group A exhibited a mean GI of 0.87, significantly lower than the 1.58 observed in Group B (*p* < 0.01).

This difference underscores the anti-inflammatory effects of *Lactobacillus reuteri*, which likely reduced the inflammatory response by modulating the oral microbiota and downregulating pro-inflammatory cytokines such as IL-1β and TNF-α.

The more significant reduction in GI in Group A indicates a more pronounced improvement in gingival health compared to the placebo group.

The mean PPD in Group A was 3.11 mm, substantially lower than the 5.30 mm recorded in Group B (*p* < 0.01).

The enhanced reduction in the PPD in Group A suggests that probiotic supplementation supported tissue healing, potentially through biofilm disruption and the competitive inhibition of pathogenic bacteria like *Porphyromonas gingivalis.*

In contrast, the limited PPD reduction in Group B aligns with the constraints of mechanical therapy alone in achieving significant tissue healing. The marked differences in the GI and PPD between the two groups underscore the role of probiotics as a valuable adjunct to standard therapy. By restoring the microbial balance and reducing inflammation more effectively, probiotics contribute to superior clinical outcomes.

The data suggest that combining probiotics with mechanical therapy offers a synergistic effect, addressing periodontal disease’s bacterial and inflammatory components.

Figure 3 provides evidence for the added value of probiotics in periodontal therapy. The significant differences in the GI and PPD between Groups A and B at T2 highlight the efficacy of *Lactobacillus reuteri* in improving clinical outcomes, reinforcing its potential as an integral component of comprehensive periodontal treatment protocols.

### 3.5. Microbiological Analysis

The levels of pathogenic bacteria (*Porphyromonas gingivalis* and *Aggregatibacter actinomycetemcomitans*) significantly decreased from T0 to T2 (*p* < 0.01). The levels of beneficial bacteria (*Lactobacillus* spp. and *Bifidobacterium* spp.) increased dramatically from T0 to T2 (*p* < 0.01). A modest reduction in pathogenic bacteria levels was observed but was not statistically significant (*p* > 0.05). The increase in beneficial bacteria levels was also not statistically significant (*p* > 0.05); see Table 5.

### 3.6. Between-Group Analysis

A comparison of Groups A and B at T2 revealed that the levels of pathogenic bacteria (*P. gingivalis* and *A. actinomycetemcomitans*) were significantly lower in Group A compared to Group B (*p* < 0.01). The levels of beneficial bacteria (*Lactobacillus* spp. and *Bifidobacterium* spp.) were significantly higher in Group A compared to Group B (*p* < 0.01); see Table 6.

Probiotic therapy combined with standard treatment generated significantly more significant clinical and microbiological improvements than standard treatment alone.

The differences between the groups were highly statistically significant (*p* < 0.0001), suggesting the efficacy of probiotics in reducing gingival inflammation and the periodontal pocket depth.

### 3.7. Changes in Inflammatory Markers

Table 7 shows that Group A demonstrated significant reductions in inflammatory markers, with IL-1β decreasing by 37% (*p* < 0.01) and TNF-α by 42% (*p* < 0.01). In contrast, Group B showed minimal changes, with 7% and 6% reductions for IL-1β and TNF-α, respectively.

## 4. Discussion

The results of this study indicate that probiotic therapy, administered as an adjunct to standard periodontal treatment, has a significantly more significant effect in terms of reducing gingival inflammation and the periodontal pocket depth than standard therapy alone. These findings are supported by the significant changes observed at both the microbiological and clinical levels.

The results of Group A revealed the significant benefits of probiotics as an adjunct to standard periodontal treatment, providing remarkable improvements in clinical and microbiological parameters.

Group A demonstrated significant improvements in gingival health and periodontal tissue healing, likely attributable to the anti-inflammatory and immunomodulatory effects of *Lactobacillus reuteri.* These findings are supported by the studies conducted by Krasse et al. (2006), which reported similar improvements after administering *Lactobacillus reuteri* [5,7].

From a microbiological perspective, Group A demonstrated a notable shift in the bacterial composition, with reduced pathogenic species and increased beneficial bacteria. This highlights the potential of probiotics to inhibit pathogenic bacteria through competition and biofilm disruption. The studies by Teughels et al. (2011) confirm that probiotics can modulate the oral microbiota, favoring recolonization with beneficial bacteria and contributing to oral health [2,5].

The results obtained in Group B show the limited effectiveness of standard therapy in the absence of a probiotic adjunct, highlighting the need for complementary strategies.

Although standard therapy showed improvements in the clinical parameters, these were less pronounced compared to Group A, reflecting the limited ability of non-surgical periodontal therapy (NSPT) to restore the oral microbiota balance and effectively address chronic inflammation.

In Group B, mechanical therapy resulted in modest changes in the bacterial composition, with a limited reduction in pathogenic species and a minimal increase in beneficial bacteria, suggesting its limited efficacy in promoting microbiota recolonization.

The timing of patient reevaluation was based on current evidence-based guidelines that suggest that significant changes in clinical and microbiological parameters occur within the first 2–3 months post-therapy. According to Paternò Holtzman et al. (2024), this timeframe allows sufficient time for the stabilization of inflammatory markers and clinical parameters such as the gingival index and periodontal pocket depth after treatment [9]. By selecting the 8-week mark post-probiotic or placebo administration, this study ensured that the outcomes measured reflected both the immediate therapeutic effects and the short-term stabilization of the periodontal parameters [9,13,14,15,16,17,18].

This phenomenon is well documented in the literature. For example, Stamatova and Meurman (2009) showed that mechanical treatments have limited effectiveness in restoring the microbiological balance of the oral cavity, which may contribute to the recurrence of inflammation and progression of periodontal disease [4,19].

The results from Groups A and B were compared to highlight the significant impact of probiotics on oral health. Group A showed a more substantial reduction in the GI and PPD, reflecting superior efficacy in controlling inflammation and tissue healing.

The significant differences in the pathogenic and beneficial bacteria levels highlight probiotics’ ability to influence the oral microbiota. These findings suggest that probiotics offer an innovative approach, complementary to standard therapy, to improving long-term clinical and microbiological outcomes in periodontal disease.

The efficacy of probiotics may also be due to their ability to regulate the host immune response. Probiotics reduce the levels of pro-inflammatory cytokines, such as IL-1β and TNF-α, thus limiting local inflammation and promoting tissue healing [10,11,12,13,14,15]. These findings agree with the meta-analysis conducted by Martin-Cabezas et al. (2016), which concluded that probiotic therapy significantly improves the effectiveness of standard periodontal treatments [14]. Previous studies have reported similar effects, highlighting the ability of probiotics to modulate the oral microbiota and promote beneficial bacteria such as *Lactobacillus* spp. and *Bifidobacterium* spp. [13,16,17,18,19]. In a study by Krasse et al. (2006), supplementation with *Lactobacillus reuteri* reduced gingival bleeding and inflammation by over 40%, confirming the benefits of this probiotic strain in oral health [7,20].

Another important observation was the significant reduction in the periodontal pocket depth (PPD) in Group A, suggesting improved tissue healing and reduced local inflammatory activity. This result is supported by a meta-analysis by Martin-Cabezas et al. (2016), which concluded that probiotic therapy can increase the effectiveness of periodontal treatment by reducing inflammation and improving clinical parameters [14].

Probiotics compete for nutrients and space to reduce the colonization of pathogenic bacteria. They also reduce the levels of pro-inflammatory cytokines such as IL-1β and TNF-α, helping to reduce inflammation [13,17,18,19,20,21].

*Lactobacillus reuteri* disrupts pathogenic biofilms, making bacteria more susceptible to standard treatments [14,22].

The results of this study demonstrate that probiotic therapy, used as an adjunct to standard periodontal treatment, significantly reduces pathogenic bacteria and increases beneficial bacteria in the oral cavity. Group A, which received *Lactobacillus reuteri* supplementation, showed a more pronounced decrease in the levels of *Porphyromonas gingivalis* and *Aggregatibacter actinomycetemcomitans* compared to Group B (placebo), confirming the potential of this probiotic strain to modulate the oral microbiota.

Moreover, there was a significant increase in the *Lactobacillus* spp. and *Bifidobacterium* spp. levels observed in Group A, supporting the idea that probiotics can promote recolonization with beneficial bacteria, essential in maintaining a healthy balance in the oral microbiota. These effects are consistent with other clinical studies, such as that conducted by Krasse et al. (2006), which reported a reduction of over 40% in gingival inflammation and bleeding with the administration of *Lactobacillus reuteri* [7].

Martin-Cabezas et al. (2016) conducted a meta-analysis that concluded that probiotics used as an adjunctive therapy could significantly reduce inflammation and improve the clinical parameters of periodontal disease, including the periodontal pocket depth and gingival index [14,23]. Similarly, the study by Vivekananda et al. (2010) showed that the administration of *Lactobacillus reuteri* in combination with standard periodontal therapy resulted in a significant reduction in pathogenic bacteria levels and marked improvements in clinical outcomes compared to standard treatment alone [19,24,25,26,27,28].

Another important aspect is the role of probiotics in regulating the host immune response. Studies have shown that *Lactobacillus reuteri* and other probiotic strains can reduce the levels of pro-inflammatory cytokines, such as IL-1β and TNF-α, thereby helping to limit periodontal tissue destruction [4,29,30,31,32,33,34].

*Lactobacillus reuteri* was selected for its well-documented anti-inflammatory and microbiota-modulating properties. Studies by Krasse et al. (2006) and Teughels et al. (2011) have shown that L. reuteri effectively reduces the levels of key periodontal pathogens, such as *Porphyromonas gingivalis*, while promoting recolonization with beneficial bacteria. Comparative analyses with other strains, including *Lactobacillus salivarius* and *Bifidobacterium* spp., suggest that L. reuteri exhibits superior efficacy in reducing gingival inflammation and the pocket depth. These properties make it an ideal candidate for adjunctive periodontal therapy [5,7].

In this context, our results extend these findings, highlighting that probiotic therapy reduces inflammation and contributes to restoring the balance of the oral microbiota, an essential component in preventing the recurrence of periodontal disease.

The findings of our study align with those reported by Martin-Cabezas et al. (2016), who demonstrated that probiotics as an adjunctive therapy significantly reduce inflammatory markers and improve clinical parameters in periodontal patients [14]. Similarly, Ausenda et al. (2023) provided robust evidence of the clinical, microbiological, and immunological benefits of various probiotic strains, including Lactobacillus reuteri, over long-term use [35]. However, unlike the present study, these meta-analyses often included a broader range of probiotic strains and extended durations [12,34].

Our study’s significant reduction in the gingival index and periodontal pocket depth corroborates these meta-analyses while emphasizing the specific efficacy of *Lactobacillus reuteri*.

This study had a relatively short follow-up period (8 weeks) and a moderate sample size (80 patients). Further studies with an extended follow-up period and a larger sample size are needed to assess the effects’ durability and investigate potential adverse effects.

The short follow-up period of 8 weeks represents a limitation in assessing the long-term sustainability of the observed clinical and microbiological improvements. The existing literature indicates that the benefits of probiotics, including *Lactobacillus reuteri*, may extend beyond this timeframe. For instance, meta-analyses such as that of Ausenda et al. (2023) underscore the importance of monitoring probiotics’ efficacy over several months to evaluate their role in preventing the recurrence of inflammation and sustaining the oral microbiota balance [35]. Future research should focus on extending the follow-up period to 6 months or longer to confirm the durability of these effects.

## 5. Conclusions

Probiotics, particularly *Lactobacillus reuteri*, represent a promising option to complement standard periodontal therapy. Our findings support their use as an integral part of periodontal treatment, with the potential to significantly improve clinical and microbiological outcomes.

## Figures and Tables

**Table 1:** Baseline characteristics of study participants.

Characteristic	Group A (Probiotics)	Group B (Placebo)
Number of participants	40	40
Age (mean ± SD)	45 ± 6 (39–51 years)	46 ± 5 (41–51 years)
Women (%)	65%	60%
Men (%)	35%	40%
Age (mean ± SD)	45 ± 6	46 ± 5
Gingival index (mean ± SD)	2.7 ± 0.9	2.6 ± 0.8
Periodontal pocket depth (mean ± SD)	5.5 ± 0.8 mm	5.6 ± 0.9 mm
Bleeding on probing (%)	80%	78%
Pathogenic bacteria presence (%)	90%	88%

**Table 2:** Changes in GI and PPD from T0 to T2 with *p*-values.

Parameter	T0	T2	Reduction	*p*-Value
Gingival Index (GI)	2.70	0.87	1.83	<0.0001
Periodontal Pocket Depth (PPD)	5.53	3.11	2.42	<0.0001

**Table 3:** Changes in GI and PPD from T0 to T2 with *p*-values (Group B).

Parameter	T0	T2	Reduction	*p*-Value
Gingival Index (GI)	2.42	1.58	0.84	<0.0001
Periodontal Pocket Depth (PPD)	5.71	5.30	0.41	<0.0001

**Table 4:** Differences between Group A and Group B at T2.

Parameter	Group A (T2)	Group B (T2)	F-Value	*p*-Value
Gingival Index (GI)	0.87	1.58	58.54	<0.0001
Periodontal Pocket Depth (PPD)	3.11	5.30	2820.31	<0.0001

**Table 5:** Level of pathogenic and beneficial bacteria in Group A and Group B.

Bacteria	Group A (T0)	Group A (T2)	*p*-Value	Group B (T0)	Group B (T2)	*p*-Value
*Porphyromonas gingivalis*	4.8 × 10⁶ CFU	1.2 × 10⁶ CFU	<0.01	4.7 × 10⁶ CFU	3.9 × 10⁶ CFU	0.08
*Aggregatibacter actinomycetemcomitans*	3.1 × 10⁵ CFU	0.9 × 10⁵ CFU	<0.01	3.2 × 10⁵ CFU	2.7 × 10⁵ CFU	0.07
*Lactobacillus* spp.	1.5 × 10⁵ CFU	4.6 × 10⁵ CFU	<0.01	1.4 × 10⁵ CFU	1.7 × 10⁵ CFU	0.10
*Bifidobacterium* spp.	2.2 × 10⁵ CFU	5.1 × 10⁵ CFU	<0.01	2.3 × 10⁵ CFU	2.5 × 10⁵ CFU	0.12

**Table 6:** Comparison of bacterial levels between Groups A and B at T2.

Bacteria	Group A (T2)	Group B (T2)	*p*-Value
*Porphyromonas gingivalis*	1.2 × 10⁶ CFU	3.9 × 10⁶ CFU	<0.01
*Aggregatibacter actinomycetemcomitans*	0.9 × 10⁵ CFU	2.7 × 10⁵ CFU	<0.01
*Lactobacillus* spp.	4.6 × 10⁵ CFU	1.7 × 10⁵ CFU	<0.01
*Bifidobacterium* spp.	5.1 × 10⁵ CFU	2.5 × 10⁵ CFU	<0.01

**Table 7:** Changes in inflammatory markers (IL-1β and TNF-α) in both groups after 8 weeks of treatment.

Inflammatory Marker	Group A (T0)	Group A (T2)	% Reduction (Group A)	Group B (T0)	Group B (T2)	% Reduction (Group B)	*p*-Value
IL-1β (pg/mL)	25.0 ± 3.1	15.8 ± 2.7	37%	24.5 ± 3.5	22.8 ± 3.2	7%	<0.01
TNF-α (pg/mL)	30.5 ± 4.0	17.7 ± 3.4	42%	29.8 ± 4.2	27.9 ± 3.8	6%	<0.01

## Data Availability

The data is available in the article.
